# Consensus Statement on Ethical & Safety Practices for Conducting Digital Monitoring Studies with People at Risk of Suicide and Related Behaviors

**DOI:** 10.1176/appi.prcp.20200029

**Published:** 2020-12-21

**Authors:** Matthew K. Nock, Evan M. Kleiman, Melissa Abraham, Kate H. Bentley, David A. Brent, Ralph J. Buonopane, Franckie Castro-Ramirez, Christine B. Cha, Walter Dempsey, John Draper, Catherine R. Glenn, Jill Harkavy-Friedman, Michael R. Hollander, Jeffrey C. Huffman, Hye In S. Lee, Alexander J. Millner, David Mou, Jukka-Pekka Onnela, Rosalind W. Picard, Heather M. Quay, Osiris Rankin, Shannon Sewards, John Torous, Joan Wheelis, Ursula Whiteside, Galia Siegel, Anna E. Ordóñez, Jane L. Pearson

**Affiliations:** Department of Psychology, Harvard University, Cambridge, Massachusetts (Nock, Bentley, Castro-Ramirez, Lee, Millner, Mou, Rankin); Department of Psychiatry, Massachusetts General Hospital, Boston, Massachusetts (Nock, Abraham, Bentley, Huffman, Mou); Franciscan Children’s, Boston, Massachusetts (Nock, Buonopane, Millner); Department of Psychology, Rutgers University, New Brunswick, New Jersey (Kleiman); Department of Clinical Research, Massachusetts General Hospital, Research Ethics Consultation Unit, Boston, Massachusetts (Abraham); Department of Psychiatry, University of Pittsburgh School of Medicine, Pittsburgh, Pennsylvania (Brent); Department of Counseling and Clinical Psychology, Teachers College, Columbia University, New York, New York (Cha); Department of Biostatistics, University of Michigan School of Public Health, Ann Arbor, Michigan (Dempsey); National Suicide Prevention Lifeline, vibrant Emotional Health, New York, New York (Draper); Department of Psychology, Old Dominion University, Norfolk, Virginia (Glenn); American Foundation for Suicide Prevention, New York, New York (Harkavy-Friedman); Department of Psychiatry, McLean Hospital, Belmont, Massachusetts (Hollander, Wheelis); Department of Biostatistics, Harvard T.H. Chan School of Public Health, Boston, Massachusetts (Onnela); Massachusetts Institute of Technology, MIT Media Lab, Cambridge, Massachusetts (Picard); Harvard University, Office of the General Counsel, Cambridge, Massachusetts (Quay); Department of IRB Administration, Harvard University, Cambridge, Massachusetts (Sewards); Department of Psychiatry, Beth Israel Deaconess Medical Center, Boston, Massachusetts (Torous); University of Washington, Behavioral Research and Therapy Clinics, Seattle, Washington (Whiteside); National Institute of Mental Health, Office of Clinical Research, Bethesda, Maryland (Siegel, Ordóñez); National Institute of Mental Health, Special Advisor to the Director on Suicide Research, Bethesda, Maryland (Pearson). National Suicide Prevention

## Abstract

**Objective:**

Digital monitoring technologies (e.g., smart-phones and wearable devices) provide unprecedented opportunities to study potentially harmful behaviors such as suicide, violence, and alcohol/substance use in real-time. The use of these new technologies has the potential to significantly advance the understanding, prediction, and prevention of these behaviors. However, such technologies also introduce myriad ethical and safety concerns, such as deciding when and how to intervene if a participant’s responses indicate elevated risk during the study?

**Methods:**

We used a modified Delphi process to develop a consensus among a diverse panel of experts on the ethical and safety practices for conducting digital monitoring studies with those at risk for suicide and related behaviors. Twenty-four experts including scientists, clinicians, ethicists, legal experts, and those with lived experience provided input into an iterative, multi-stage survey, and discussion process.

**Results:**

Consensus was reached on multiple aspects of such studies, including: inclusion criteria, informed consent elements, technical and safety procedures, data review practices during the study, responding to various levels of participant risk in real-time, and data and safety monitoring.

**Conclusions:**

This consensus statement provides guidance for researchers, funding agencies, and institutional review boards regarding expert views on current best practices for conducting digital monitoring studies with those at risk for suicide—with relevance to the study of a range of other potentially harmful behaviors (e.g., alcohol/substance use and violence). This statement also highlights areas in which more data are needed before consensus can be reached regarding best ethical and safety practices for digital monitoring studies.

The development of new technologies such as smart-phones and wearable biosensors has provided unprecedented opportunities to study a wide range of mental health concerns and to improve their understanding, prediction, and prevention. The newfound ability to monitor people’s thoughts, affect, and behavior in real-time has the potential to significantly advance the understanding of potentially harmful behaviors that occur episodically in natural environments and to develop new just-in-time adaptive interventions (1) to help mitigate them. Perhaps the most concerning of such clinical high-risk behaviors are suicide and its immediate precursors—suicidal thoughts and behaviors (STBs). Studies using digital monitoring of those at risk for STBs are increasing exponentially in number in recent years (2). However, these newly available methods also present unprecedented scientific, methodological, clinical, ethical, and legal concerns. There has been no consensus among scientists, clinicians, and other stakeholders about best practices for conducting real-time monitoring studies of those at risk for suicide and related behaviors, leaving these parties on their own to work through these issues and propose solutions.

Prior studies have provided valuable guidance for researchers, funding agencies, and institutional review boards (IRBs) in planning and carrying out research with those at risk for suicide (3–5). Digital monitoring methods present a host of new factors for consideration. Whereas traditional assessment approaches ask participants to retrospectively report on the presence, characteristics, and risk of STBs over a period of weeks or months at a time, digital monitoring approaches and studies assess such outcomes *at that very moment* at which they occur and can do so repeatedly over periods of minutes, hours, days, and weeks. Moreover, some digital monitoring apps also allow the passive collection of data on participants’ exact geo-location (e.g., GPS). This means that digital monitoring studies often can tell us not only *when* someone is at risk, but *where* someone is at the time of risk. Given these considerations, funding agencies, IRBs, and researchers commonly raise important participant safety and privacy questions to be considered and decided before, during, and after the conduct of such studies. For instance, what should researchers do when a participant’s response suggests that they are at high or imminent risk for suicide at that very moment? Is an automated response sufficient to manage participant risk, or should there be a follow-up by phone, text or e-mail by a member of the research team to conduct a more thorough risk assessment to ensure appropriate and timely treatment?

Here we report on a consensus meeting that included leading scientists, clinicians, ethicists, legal experts, those with lived experience, and other interested stakeholders regarding the safe and ethical conduct of digital monitoring studies of those at risk for suicide and related behaviors. Our goals were to outline areas of consensus to provide guidance for those using digital monitoring to study suicidal and related behaviors, and to discover and discuss areas where there is not yet consensus to point toward key questions for future research on this topic.

## METHODS

### Delphi Process

We used a modified Delphi process to obtain expert opinions about the conduct of digital monitoring studies with those at risk for suicide and related behaviors. This approach has been used to obtain expert opinion and consensus across the medical and social sciences in areas where none yet exists (6–9). Essential elements of the Delphi process include: assembling a team of experts representing a broad range of perspectives, receiving iterative and anonymous assessments from these experts to obtain their candid and unbiased input, and group discussion of key issues facilitated by review of the anonymous and aggregated results to allow the group to consider all perspectives before final assessment of individual experts’ views on the key issues.

#### Identification of key issues/questions on this research topic.

The first step in this modified Delphi process was to generate a list of key issues and questions on this research topic about which expert consensus was lacking. This was done via a thorough literature review followed by a series of discussions between two university-based clinical scientists (Matthew K. Nock and Evan M. Kleiman) and National Institute of Mental Health (NIMH)-based scientists (Jane Pearson, Galia Siegel, and Anna Ordóñez) who planned and organized the rest of the process. Our approach was to cast a broad net and to generate issues/questions at each phase of the research process (outlined below).

#### Panel selection.

The second step was to generate a diverse list of experts who could provide guidance on the range of issues/questions produced. The experts invited to participate represent the perspectives of those working in the areas of: science, clinical practice (inpatient/outpatient and child/adolescent/adult), bioethics/legal/IRB, computer science, statistics, funding agencies (federal and private), and those with personal/lived experience with STBs. We invited 23 experts to participate in this process. To increase the diversity of perspectives included, we also invited four post-doctoral and two pre-doctoral research fellows, each of whom had multiple years of experience working in this area, for a total of 29 invitees.

#### Survey: Round 1.

The third step was to develop and send a 19-item survey to all invited participants. These questions focused on two time periods: (1) issues to consider before data collection begins (described in detail in Table 1) and (2) issues to consider during data collection (described in detail in Table 2). Consistent with suggested guidelines for Delphi surveys, (8, 9) this survey was conducted anonymously to get participants’ unbiased responses to each question. When possible, questions included response options that could be easily quantified (e.g., no/yes, check all that apply) to facilitate efforts to reach a consensus. All questions also included an open-ended response option so that participants could provide more nuanced responses if the response options did not adequately capture their perspective. Administration of this survey and all procedures described here were approved by the Harvard University IRB. An anonymous link to the survey was e-mailed to all 29 invitees. Twenty-four (82.8%) participants completed the survey.

#### Consensus meeting.

In the fourth step, all invitees were invited to attend a day-long, face-to-face meeting co-hosted by researchers from Harvard University and NIMH. Twenty-seven of the 29 invitees attended (93.1%). Each invitee presented information about their experience and/or perspectives in this area. We then shared the aggregated results of the Survey: Round 1 and talked through each topic assessed in the survey in detail.

#### Survey: Round 2.

In the fifth and final step in this process, we modified the survey based on the previous round of responses (e.g., clarifying questions in places where there was confusion about what was being asked) and on the discussion at the face-to-face meeting. The updated survey re-administered the first 13 items of the prior survey (because we had complete consensus on the final 6 items) with several modifications. We sent this version of the survey to the 27 people who participated in the face-to-face meeting (and had the benefit of the extended discussion about these issues), of whom 21 (77.8%) participants completed the survey.

### Data Analysis

Here we report the results from each of the final questions asked of this panel of experts. There currently is no agreed upon standard regarding what constitutes a “consensus” using the Delphi process (irony noted: no consensus on consensus). Given the standard convention in the social and health sciences for considering agreement of 70% or higher to represent an acceptable level of agreement for inter-rater reliability, response rates, validity, and so on, we considered agreement of 70% or higher to represent “agreement” (i.e., consensus) and 80% or higher to represent “strong agreement.”

## RESULTS

### Issues to Consider Before Data Collection Begins

The panel of experts raised and discussed a range of issues that encompass decisions about, and interactions with, potential study participants before data collection begins. These included issues regarding who to enroll in a study, under what conditions, what information to include in the informed consent process, how much contact information to collect from participants, and what technical and safety procedures to set in place.

#### Exclusion of participants.

The first question addressed by the panel was whether any potential participants should be excluded from real-time monitoring studies due to elevated risk of suicide. There was strong agreement that researchers should aim to collect data from those even at the highest levels of risk, and thus that potential participants should not be excluded solely due to high level of suicide risk. This perspective was endorsed by 19/21 experts (90.5%). Two people (9.5%) thought that those at very high risk of STBs should be excluded because they might be “unable to agree to seek crisis care” or “unable to respond to ecological momentary assessment prompts.”

#### Conditions for enrolling in the study.

There also was strong agreement (85.7% of experts) that there should not be any conditions that participants must agree to in order to enroll in a real-time monitoring study, such as agreeing to be in treatment or call a hotline when at high risk. Experts also indicated that such contingencies would be difficult to enforce, would not increase participant safety, and seem intended to protect the researcher/institution more than the participant. The 14.3% of experts who indicated that participants should agree to some conditions to enter or remain in the study all endorsed that those should include: (a) agreeing to call a hotline or seek other help when at high risk, (b) agree to access a clinician when at imminent risk, and (c) agree to provide data with some frequency. At least one expert also endorsed each of the following: agreeing to have a safety plan, providing collateral contact information, and getting permission from their current clinician (if they have one) to participate.

#### Informed consent.

The panel reached consensus on 10 elements that should be conveyed to participants during the consent process (i.e., written in the consent form and conveyed to the participant verbally when possible). There was strong consensus for nine of these elements (Table 1), with consensus, but some divergence of opinion, regarding whether participants should be informed about what risk monitoring activities and interventions may be taking place in the study. Some experts believed that efforts to monitor and intervene during high-risk situations may be compromised if participants are made aware of the specifics of potential monitoring approaches or interventions, whereas others did not share this concern.

#### Contact information.

There was strong agreement that investigators conducting digital monitoring studies should have some method of contacting participants in the event of elevated risk, including: participant phone number, parent contact information (in the case of child/adolescent participants), home address, and email address. There also was agreement that investigators should obtain contact information for at least one collateral person who could help reach the participant in times of increased risk. There was not a consensus on a requirement for multiple collaterals, participants’ clinicians’ contact information, or having access to participants’ social media accounts as a means of communicating. Two notable exceptions to the need for participant or collateral contact information are studies of hospital inpatients and those recruited anonymously online. In the former, hospital staff should be alerted if a participant reports elevated risk. In the latter, referrals to higher levels of care may be made in an automated message/referral.

#### Technological and safety procedures.

There was strong agreement on seven different technological and safety procedures that investigators should conduct before beginning a digital monitoring study in this area (Table 1). Several experts suggested that investigators should solicit feedback from participants about their desired response from the investigative team for various levels of risk; however, there was not a consensus on this point, as other experts did not believe that having individual-level risk responses is feasible for larger studies.

### Issues to Consider During Data Collection

The most challenging aspects of conducting digital monitoring research with people at elevated risk for suicide and related behaviors involve determining when and how to monitor participant data and manage suicide risk. The panel of experts spent most of its time discussing issues in this area, including how frequently to check participant data, how quickly to respond to those at elevated risk, how to define and respond to elevated risk, as well as issues of study monitoring and data security.

#### Frequency of reviewing participant data.

Investigators may have access to software platforms that allow for continuous, real-time monitoring of study data. In some instances, real-time monitoring of data is not possible (e.g., a survey app does not have the ability to automatically alert the research team when a participants’ response crosses a specified threshold, or if data are only uploaded when a Wi-Fi connection is available). Regardless, the panel agreed that the research team should have a protocol specifying the frequency with which a team member would check the data for high-risk responses. There was strong agreement (89.5%) that in such instances data should be reviewed at least every weekday (Table 2). There was not consensus regarding how frequently data should be reviewed within shorter windows.

#### Determining risk level.

Much of the in-person meeting was spent discussing how to determine a participant’s level of current risk (e.g., low, moderate, high, and imminent) at a given assessment point. Although there was no consensus on how to do this—as is the case with suicide risk assessment more generally (10)—there was strong agreement on the pieces of information that researchers should collect to determine risk level: current level of desire to die, level of intent to die, presence of a suicide plan, and access to the planned method. Importantly, the group noted that self-report of each of these aspects of suicidal thinking/intention is not necessarily indicative of high probability of suicidal behavior; determining a given participant’s probability of suicidal behavior at a given point in time is one of the desired goals of research of this type. In the meantime, self-reported level of suicidal thinking/intention is used as a best estimate of participant level of risk.

#### Length of response window.

Once the research team receives indication that a participant is at “imminent risk,” how quickly should they respond? The simple answer is “as quickly as possible.” However, what if the indication comes at 2 a.m. or while the team member responsible for responding is conducting a therapy session or otherwise not monitoring their phone or computer for messages? There was strong consensus (94.4%) that the team should respond within 24 h of receiving an “imminent risk” indication, and consensus (72.2%) that such a response should be made within 12 h. For instance, a team may not have the resources (e.g., person-power) to monitor responses overnight (e.g., 9 p.m.–9 a.m.), but in such a case should respond no longer than 12 h after the imminent risk indication was received.

#### Potential interventions.

The majority of experts (>61.1%) believed that for those currently at low/moderate risk, an automated message suggesting that the participant contact a crisis line, support person, or clinician is a sufficient response by the research team. For those at high risk, there was consensus that participants should receive a personalized safety plan and/or an automated additional risk assessment (77.8%), and some experts suggested that best practice should be to always have a safety plan (and associated call numbers) readily available to participants within the survey app. There was strong consensus that in cases of high risk the research team should reach out to the participant directly to conduct a risk assessment (94.4%). There was not consensus about the need to call 911 to request a wellness check, with 50% of experts endorsing such an action. Some experts suggested this “should be the last possible option” and noted potential negative consequences to doing so in cases of working with potentially vulnerable participants, such as racial/ethnic minority participants, who experience significantly higher rates of physical force and death in police-initiated contacts (11, 12).

The group also discussed the extent to which an automated intervention (e.g., pop-up with safety plan or one-touch call to clinician or hotline) versus human outreach (e.g., call, text, or email from member of the research team) should be used. There was strong consensus that automated interventions are sufficient for low risk and moderate risk situations, but that human outreach is preferred for high-risk situations and in studies of minors. Experts noted that automated outreach is sufficient in studies where participation is anonymous. There was strong consensus that researchers should formulate a personalized risk plan with each participant before the monitoring portion of the study begins to guide procedures for responding to instances in which participants do not respond to initial calls, texts, or emails from the research team (Table 2). There also was strong consensus that in studies involving youth, a parent should be contacted in instances of non-response by high-risk adolescents.

#### Participant removal.

An important question in the current context is whether participants should be removed (temporarily or permanently) from the study by the research team (vs. an individual participant’s wish to discontinue), due to elevated risk of suicide or clinical severity or worsening. There was strong consensus among experts that no participants should be removed from real-time monitoring studies—as it is important to understand and be able to predict harmful behavior among those at all levels of risk, particularly those at highest risk.

#### Data safety and monitoring.

Data and Safety Monitoring Boards (DSMBs) and Independent Safety Monitors (ISMs) often are required for NIMH-supported grants.nih.gov/grants/glossary.htm” title=”http://grants.nih.gov/grants/glossary.htm”>clinical trials “to assure the safety of research participants, regulatory compliance, and the data integrity,” (3, 13) but could be used in any study. One of their main functions is to review adverse events. There was not consensus on the requirement for DSMBs or ISMs in the case of real-time monitoring assessment studies. Approximately one-third (31.6%) of experts indicated that they are not needed for such studies, one-half (47.4%) indicated they are needed but only for studies where participants are recruited because they are at high risk, and a smaller percentage (21.1%) indicated they should be required of all such studies. For those indicating that such studies should have a DSMB/ISM, there was strong consensus that there should be a requirement that such oversight is provided by someone with expertise managing suicide risk (Table 2).

#### Data security.

There was strong consensus that researchers conducting such research use secure web-based platforms and de-identified data storage, and consensus that HIPAA compliant platforms also should be used in such research.

## DISCUSSION

Digital monitoring technologies provide unprecedented opportunities to advance the understanding of suicide and related behaviors. However, there are not yet accepted guidelines regarding ethical and safety practices for conducting research studies in this area. We convened a panel of experts to attempt to reach consensus about key considerations in this area based on currently available data and perceived best practices. This panel of experts reached consensus on a number of key issues regarding ethical and safety practices for conducting digital monitoring studies with those at high clinical risk (Table 3). Many of these aspirations reflect the need to expand study inclusion to participants with significant suicide risk, since historically they have been typically excluded from research. In many cases these aspirations also reflect the need for significant resources to enable researchers to conduct intensive longitudinal monitoring and real-time risk management and intervention, which is not always possible. This consensus statement can help to guide researchers, funding agencies, and IRBs involved in the planning, conduct, and oversight of research studies in this area. Notably, the panel of experts discussed and endorsed the fact that these views are based on currently available data and thinking, and that these views may evolve over time as additional data and considerations become available—highlighting the need to revisit these ethical and safety considerations iteratively over time.

There also were several areas in which the panel did not reach consensus. These included: (a) determination of what constitutes low, moderate, high, and imminent risk for suicide and related behaviors; (b) determination of the most effective intervention for each risk level; and (c) the requirements for using DSMBs and ISMs for monitoring studies of this type. Reaching consensus on the first two areas requires additional empirical data. Historically, determinations about level of risk for suicide and related behaviors and the most appropriate response (e.g., hospitalization) have been made primarily based on clinical judgment and decision-making. Recent advances in electronic record-keeping and machine learning have significantly enhanced the ability to predict suicidal and related behaviors, (14–16) and similar innovations are needed in studies of real-time digital monitoring. Still absent from all such studies are data on the most effective intervention for a particular person at a particular time point—a long-standing question in mental health research and practice (17). Efforts to address questions such as this are currently underway (18, 19) and hopefully will provide guidance that can be used to update the current consensus at that time.

This panel of experts considered, but did not sufficiently discuss, several related topics that require further attention. These include further incorporation of research participants’ perspectives; consideration of issues unique to children and adolescents; issues unique to other behaviors such as substance use (e.g., if/how to intervene if the research team believes a person may operate an automobile while intoxicated?); and issues unique to other digital platforms such as social media apps. The current effort represents a key step toward providing consensus guidance on safety and ethical practices for conducting digital monitoring studies with those at high clinical risk. Additional, iterative efforts are needed to provide ongoing guidance on these important topics.

## Figures and Tables

**TABLE 1.** Issues to consider before data collection begins in digital monitoring studies of those at risk for suicide and related behaviors

Issue	Question	Answer	
Exclusion of participants	Should any potential participants be excluded due to elevated risk of suicide? (Select one)	No, we should be collecting data from everyone, even those at the highest levels of suicide risk	90.5%
		Yes, people who are too high risk should not participate in research	9.5%
Conditions to staying in the study	Should there be certain conditions participants must agree to in order to enter/stay in a study, such as agreeing that they will go to treatment sessions or call a hotline when at high risk? (Select one)	No, we can encourage people to do these things but should not set any such conditions	85.7%
		Yes, there should be conditions participants must agree to enter/stay in the study	14.3%
Informed Consent	Should participants be explicitly informed of the following during informed consent? (Select all that apply)	Whether responses can trigger follow-up and/or intervention actions by the research team and/or clinicians, which may include breaking of confidentiality	100%
		Information that the participant should not rely on the study monitoring to keep them safe/alive	100%
		Information about who will have access to their data including third party software developers to improve their app product	100%
		How often researchers will check participants’ responses	95.2%
		Circumstances under which the subject’s participation may be terminated by the investigator without regard to the subject’s consent	95.2%
		Who to contact in case of crisis	95.2%
		Information that there can be technology failures	95.2%
		Information that the participant won’t be automatically hospitalized if their responses trigger a follow up assessment by the research team	90.5%
		How and what information will be shared with participants and others if confidentiality is breached	90.0%
		What risk monitoring activities and interventions will be taking place	76.2%

Issue	Question	Answer	Adults	Adolescents
Contact information	What contact information, including collateral contact information, should studies be required to collect? (Select all that apply)	Participant cell phone number	100%	95.2%
		Parent contact information	-	90.5%
		Participant home address	88.9%	76.2%
		Participant email address	83.3%	71.4%
		Collateral contact	72.2%	61.9%
		Multiple collateral contacts	38.9%	33.3%
		Participant’s clinician	27.8%	28.6%
		Participant’s social media account(s)	11.1%	9.5%
		None	14.3%	0%

Issue	Question	Answer	
Technological and safety procedures	Which issues should researchers address BEFORE data collection begins in a real-time monitoring study?		
	Technology (select all that apply)	Figure out what to do when technology fails	100%
		Test the alert system	100%
		In a standardized manner, go over items of incidental data collection (medical information, voice, video) being obtained and the process of alerting and collecting data	100%
		Determine how to store and potentially share location data	95.2%
	Safety (select all that apply)	Provide the participant with emergency contact information	100%
		Determine what criteria should be used to delineate specific criteria for triggering further risk assessment or intervention	100%
		Train study staff with risk assessment protocol for assessing and responding to participant suicidal ideation and behavior	100%
		Solicit feedback from participant about their desired response from research team for varying levels of risk	55.0%

**TABLE 2.** Issues to consider during data collection in digital monitoring studies of those at risk for suicide and related behaviors

Issue	Question	Answer	
Frequency of reviewing participant data	If available technology is not able to alert researchers in real time, how often should participant data be reviewed by a human on the research team for risk assessment purposes? (Select one)	More than two times a day	5.3%
		Twice every day	21.1%
		Once every day	31.6%
		Every weekday	31.6%
		Less than once a day	10.5%
Determining risk level	What key pieces of information should researchers collect to determine a participant’s level of risk? (Select all that apply)	Level of intent to die	94.4%
		Presence of suicide plan	94.4%
		Access to suicide plan/method	88.9%
		Level of desire to die	83.3%
		Presence of any suicidal ideation (no/yes)	66.7%
Length of response window	When the research team learns that the participant is at “imminent risk,” what is the longest acceptable time window to respond? (Select one)	Within 6 h	22.2%
		Within 12 h	50.0%
		Within 24 h	22.2%
		Within 48 h	0%
		Within 72 h	5.6%

			Risk level	
Issue	Question	Answer	Low/Moderate	High
Potential interventions	What action/intervention should be taken in each case? (Select all that apply)	Pop up message with suggestions to call a suicide hotline/crisis line	66.7%	66.7%
		Pop up message with suggestion to contact participant’s supports such as family	66.7%	50.0%
		Pop up message with suggestion to call clinician	61.1%	61.1%
		Pop up message with personalized safety plan instructions	61.1%	77.8%
		Pop up automated/interactive risk assessment	44.4%	77.8%
		Research team contacts participant for risk assessment	11.1%	94.4%
		Research team calls 911 to request wellness check	5.5%	50.0%

Issue	Question	Answer	Automated is sufficient	Human outreach needed
	At what level is an automated intervention sufficient and at which level of risk is human out-reach required? (Select one option for each row)	Low risk	100%	0%
		Moderate risk	80.0%	20.0%
		High/imminent risk	20.0%	80.0%

Issue	Question	Answer	Adult	Adolescent
Steps for reaching out to participants	If you believe that human outreach is required, what steps should be taken if a participant is not reachable by the research team? (select all that apply)	Formulate a personalized risk plan beforehand that is agreed upon during consent process	85.0%	80.0%
		Notify parent	20.0%	90.0%
		Notify collateral contact	65.0%	65.0%
		Contact clinician	20.0%	30.0%
		Call 911 and send ambulance team to participant	30.0%	30.0%
		Do nothing	5.0%	5.0%

Issue	Question	Answer	
Participant removal	Should participants be removed from the study due to elevated risk or clinical severity? (Select one)	No, we should be collecting data from everyone. Even those at the highest levels of risk	100%
		Yes, people who are too high risk should not participate in research	0%
Data and safety monitoring boards & independent safety monitors	Should all real-time monitoring studies have a DSMB or ISM? (Select one)	No, DSMBs or ISMs are not needed for such studies	31.6%
		Yes, but DSMBs or ISMs should be required only for such studies with participants at high risk for suicide	47.4%
		Yes, DSMBs or ISMs should be required for all such studies	21.1%
DSMB/ISM members	What DSMB members should be required for real-time monitoring type studies? (Select all that apply)	Expert in managing suicide risk	91.7%
		Someone with an understanding of the technology	58.3%
		Licensed clinician	41.7%
		Community member (someone with lived experience)	33.3%
		Legal expertise in the field of psychiatry	25.0%
Data security	What are minimal standards for data security? (Select all that apply)	Secure web-based platforms	94.4%
		Deidentified data storage	83.3%
		HIPAA compliant platform	77.8%

Abbreviations: DSMB, Data and Safety Monitoring Boards; ISM, Independent Safety Monitor.

**TABLE 3.** Main points of consensus in digital monitoring studies of those at risk for suicide and related behaviors

Researchers conducting real-time monitoring studies of those at risk for suicide and related behaviors should strive to: Not exclude participants soley on the basis of elevated clinical severity or suicide risk. Not exclude or remove participants who are not willing or able to meet pre-specified conditions for participant or help-seeking (e.g., remaining in treatment or calling a hotline when at high risk). Provide participants with explicit information about key elements of study procedures during the informed consent process. Collect and retain (during the real-time monitoring period) contact information (phone, email, and home address) from both the participant and at least one collateral to facilitate contacting participants during periods of perceived elevated risk. Address key aspects of technology use and participant safety before proceeding with data collection. Review participant survey responses at least once every weekday. Respond to those determined to be at “imminent risk” for suicide within 12 h of learning of this risk. Collect data about suicidal desire, intent, plan to determine participants’ level of risk. Respond to participants determined to be at high or “imminent” risk for suicide with automated risk assessments, safety plans, and human outreach (depending on risk and type of study) as soon as possible. Store data in de-identified form, in secure servers, and in compliance with HIPAA guidelines. In cases in which data safety and monitoring boards are used they should include at least one person with expertise managing suicide risk.

## HIGHLIGHTS

- Digital monitoring technologies provide unprecedented opportunities to study potentially harmful behaviors such as suicide, violence, and alcohol/substance use in real-time, but also introduce myriad ethical and safety concerns
- We convened a panel of expert scientists, clinicians, ethicists, legal experts, and those with lived experience provided input into an iterative, multi-stage survey and discussion process
- This study provides guidance for researchers, funding agencies, and institutional review boards regarding expert views on current best practices for conducting digital monitoring studies with those at risk
